# Supplementary material for: Influence of follow-up, screening age, interval, and compliance on overdiagnosis of ductal carcinoma in situ (DCIS): A modelling study
Source: PLoS One. 2026 Jan 23;21(1):e0331821. doi: 10.1371/journal.pone.0331821 (PMC12829814; doi:10.1371/journal.pone.0331821)
Supplement: S1 File — (DOCX) [file pone.0331821.s002.docx]

**S1 File. Overdiagnosis estimation: calculation main outcomes**

Abbreviations:

SD = screen-detected,

CD = clinically detected,

IBC = DCIS progressed to invasive breast cancer

DCIS = ductal carcinoma in situ.

**S1.1. Diagnosed DCIS**

$$Diagnosed =Screendetected+Clinically detected+DCIS progressed to IBC$$

*** in scenario without screening the formula reduces to CD + IBC.**

**S1.2. Number of overdiagnosed DCIS**

$$Overdiagnosed = Diagnosed in screen scenario-Diagnosed in no screen scenario = \left[ SD+CD+IBC \right]screen-\left[ CD+IBC \right]no screen$$

**S1.3. Rate of overdiagnosed DCIS**

$$Overdiagnosis rate =\frac{Number of overdiagnosed DCIS}{Number of screened women}*100,000= \frac{\left[ SD+CD+IBC \right]screen-\left[ CD+IBC \right]no screen}{Number of mammograms}*100,000$$

**S1.4. Proportion of overdiagnosed DCIS**

$$Overdiagnosed proportion =\frac{Number of overdiagnosed DCIS}{Diagnosed DCIS screen scenario}*100\%= \frac{\left[ SD+CD+IBC \right]screen-\left[ CD+IBC \right]no screen}{\left[ SD+CD+IBC \right]screen} *100\%$$
