# Supplementary material for: Influence of follow-up, screening age, interval, and compliance on overdiagnosis of ductal carcinoma in situ (DCIS): A modelling study
Source: PLoS One. 2026 Jan 23;21(1):e0331821. doi: 10.1371/journal.pone.0331821 (PMC12829814; doi:10.1371/journal.pone.0331821)
Supplement: S1 Table — (DOCX) [file pone.0331821.s003.docx]

**S1 Table. Follow-up time and DCIS overdiagnosis**

| *Overdiagnosis rate /100.000*  Follow-up time | DCIS Grade | | | |
| --- | --- | --- | --- | --- |
|  | All | 1 | 2 | 3 |
| 2 years | 48.6 | 10.9 | 19.0 | 18.7 |
| 3 years | 46.0 | 10.4 | 17.8 | 17.8 |
| 4 years | 43.8 | 10.1 | 17.0 | 16.7 |
| 5 years | 42.2 | 9.8 | 16.3 | 16.1 |
| 10 years | 39.0 | 9.1 | 15.0 | 15.0 |
| 15 years | 38.2 | 8.8 | 14.7 | 14.7 |
| 20 years | 38.1 | 8.8 | 14.7 | 14.7 |
| 25 years | 38.1 | 8.8 | 14.7 | 14.7 |

Overall overdiagnosed DCIS rate (per 100,000 screened women) stratified by grade for a follow-up time of 2 to 25 years in Dutch screening setting (biennial, age 50-74, 76% compliance).
