# Supplementary material for: Influence of follow-up, screening age, interval, and compliance on overdiagnosis of ductal carcinoma in situ (DCIS): A modelling study
Source: PLoS One. 2026 Jan 23;21(1):e0331821. doi: 10.1371/journal.pone.0331821 (PMC12829814; doi:10.1371/journal.pone.0331821)
Supplement: S2 Table — (DOCX) [file pone.0331821.s004.docx]

**S2 Table. Screen start age and DCIS overdiagnosis: age 40-49**

| Screen at age (years)^a^ | Follow-up time (years) | | | | | | | |
| --- | --- | --- | --- | --- | --- | --- | --- | --- |
|  | 2 | 3 | 4 | 5 | 10 | 15 | 20 | 25 |
| Overdiagnosis rate (per 100,000 screened women) | | | | | | | | |
| 40 | 19.2 | 16.8 | 15.3 | 14.0 | 7.6 | 6.7 | 6.3 | 6.1 |
| 42 | 24.5 | 21.0 | 18.3 | 15.4 | 9.2 | 8.2 | 7.2 | 7.1 |
| 44 | 25.3 | 20.1 | 17.7 | 14.5 | 8.6 | 7.8 | 7.1 | 7.0 |
| 46 | 33.2 | 27.2 | 22.6 | 19.9 | 14.1 | 11.0 | 10.6 | 10.6 |
| 48 | 35.5 | 30.4 | 27.5 | 24.2 | 15.7 | 12.6 | 12.3 | 12.1 |
| Proportion overdiagnosed (per detected DCIS in screened population) | | | | | | | | |
| 40 | 42.9% | 31.1% | 23.4% | 19.2% | 4.8% | 1.5% | 0.8% | 0.6% |
| 42 | 42.2% | 33.2% | 25.3% | 18.5% | 3.7% | 1.5% | 0.9% | 0.6% |
| 44 | 42.9% | 29.8% | 21.6% | 15.3% | 2.4% | 1.2% | 0.8% | 0.6% |
| 46 | 43.9% | 31.7% | 19.7% | 12.2% | 3.0% | 1.5% | 1.0% | 0.8% |
| 48 | 34.7% | 20.5% | 13.5% | 9.4% | 2.8% | 1.5% | 1.1% | 0.9% |

DCIS overdiagnosis rate (per 100,000 women screened) and proportion overdiagnosed DCIS after a single screen at age 40-49 years for a follow-up time of 2 to 25 years in Dutch screening setting (biennial mammography, 76% compliance).
