# Supplementary material for: Influence of follow-up, screening age, interval, and compliance on overdiagnosis of ductal carcinoma in situ (DCIS): A modelling study
Source: PLoS One. 2026 Jan 23;21(1):e0331821. doi: 10.1371/journal.pone.0331821 (PMC12829814; doi:10.1371/journal.pone.0331821)
Supplement: S3 Table — (DOCX) [file pone.0331821.s005.docx]

**S3 Table. Screen start age and DCIS overdiagnosed proportion by DCIS grade**

| Screen at age (years)^a^ | Follow-up time (years) | | | | | | | |
| --- | --- | --- | --- | --- | --- | --- | --- | --- |
|  | 2 | 3 | 4 | 5 | 10 | 15 | 20 | 25 |
| Proportion overdiagnosed (per detected DCIS in screened population) | | | | | | | | |
| DCIS Grade 1 | | | | | | | | |
| 50 | 54.9% | 44.0% | 32.1% | 25.2% | 10.2% | 6.2% | 4.7% | 3.9% |
| 52 | 52.3% | 41.1% | 34.9% | 29.3% | 12.7% | 8.8% | 6.5% | 5.3% |
| 54 | 55.5% | 45.9% | 37.1% | 31.7% | 18.0% | 11.7% | 8.8% | 7.3% |
| 56 | 54.2% | 45.2% | 37.7% | 30.8% | 16.6% | 10.6% | 7.9% | 6.6% |
| 58 | 52.5% | 42.6% | 36.5% | 32.1% | 16.6% | 10.5% | 7.9% | 6.9% |
| 60 | 53.5% | 46.0% | 37.6% | 32.8% | 15.1% | 10.1% | 7.5% | 6.8% |
| 62 | 62.8% | 54.0% | 43.4% | 37.6% | 17.7% | 11.6% | 9.4% | 8.7% |
| 64 | 58.5% | 49.9% | 41.0% | 34.4% | 17.3% | 11.4% | 9.9% | 9.0% |
| 66 | 56.3% | 46.5% | 37.4% | 31.5% | 16.2% | 11.4% | 10.1% | 9.6% |
| 68 | 54.7% | 46.0% | 38.4% | 33.5% | 17.8% | 13.7% | 12.1% | 11.8% |
| 70 | 56.2% | 48.0% | 39.1% | 33.1% | 18.8% | 15.1% | 13.8% | 13.5% |
| 72 | 58.6% | 49.2% | 40.5% | 34.5% | 21.7% | 17.1% | 16.0% | 15.8% |
| 74 | 57.1% | 47.5% | 41.8% | 36.6% | 24.9% | 19.9% | 19.2% | 19.0% |
| DCIS Grade 2 | | | | | | | | |
| 50 | 45.3% | 32.0% | 21.8% | 17.9% | 7.2% | 4.4% | 3.1% | 2.4% |
| 52 | 39.5% | 31.2% | 24.7% | 20.0% | 8.7% | 5.3% | 3.8% | 2.9% |
| 54 | 49.3% | 39.7% | 32.7% | 25.8% | 11.4% | 6.9% | 4.9% | 4.0% |
| 56 | 50.1% | 38.8% | 30.5% | 24.2% | 11.0% | 6.5% | 4.7% | 4.0% |
| 58 | 47.1% | 36.4% | 29.4% | 24.2% | 9.1% | 5.4% | 4.0% | 3.6% |
| 60 | 47.5% | 37.7% | 28.6% | 23.1% | 9.4% | 5.7% | 4.4% | 4.1% |
| 62 | 48.7% | 37.7% | 29.2% | 23.1% | 9.0% | 5.6% | 4.7% | 4.4% |
| 64 | 49.0% | 38.0% | 29.1% | 23.3% | 10.2% | 6.6% | 5.7% | 5.4% |
| 66 | 46.9% | 36.3% | 27.6% | 22.9% | 10.8% | 7.8% | 7.1% | 6.8% |
| 68 | 44.9% | 36.6% | 29.9% | 23.9% | 11.7% | 9.0% | 8.4% | 8.2% |
| 70 | 49.9% | 38.9% | 31.3% | 24.3% | 12.6% | 10.5% | 9.8% | 9.6% |
| 72 | 52.8% | 40.6% | 33.7% | 27.8% | 16.6% | 14.4% | 13.8% | 13.6% |
| 74 | 51.2% | 41.6% | 34.9% | 29.6% | 20.0% | 17.6% | 16.7% | 16.7% |
| DCIS Grade 3 | | | | | | | | |
| 50 | 42.0% | 31.9% | 22.4% | 16.8% | 6.0% | 3.3% | 2.3% | 1.8% |
| 52 | 41.5% | 30.4% | 23.7% | 18.7% | 7.3% | 4.1% | 2.9% | 2.2% |
| 54 | 45.1% | 34.9% | 27.8% | 21.4% | 8.9% | 5.4% | 4.0% | 3.3% |
| 56 | 47.0% | 34.7% | 27.3% | 21.8% | 8.7% | 5.4% | 4.0% | 3.5% |
| 58 | 44.5% | 33.7% | 25.8% | 20.0% | 7.8% | 4.8% | 3.6% | 3.3% |
| 60 | 44.7% | 33.4% | 25.5% | 19.9% | 8.7% | 5.6% | 4.5% | 4.3% |
| 62 | 42.3% | 31.1% | 23.6% | 19.2% | 8.6% | 5.1% | 4.3% | 4.1% |
| 64 | 42.8% | 33.2% | 25.9% | 20.2% | 8.9% | 5.5% | 4.9% | 4.7% |
| 66 | 45.9% | 34.7% | 26.9% | 22.1% | 9.1% | 6.6% | 5.9% | 5.7% |
| 68 | 42.9% | 34.1% | 27.2% | 21.1% | 8.6% | 6.8% | 6.2% | 6.1% |
| 70 | 48.6% | 37.2% | 27.9% | 21.5% | 11.1% | 9.4% | 8.9% | 8.7% |
| 72 | 46.5% | 34.3% | 27.2% | 22.8% | 13.6% | 11.6% | 11.1% | 10.9% |
| 74 | 42.5% | 34.2% | 26.4% | 22.9% | 15.1% | 13.0% | 12.6% | 12.5% |

Proportion overdiagnosed DCIS after a single screen at age 50-74 years for a follow-up time

of 2 to 25 years in Dutch screening setting (biennial mammography, 76% compliance).
