# Supplementary material for: Influence of follow-up, screening age, interval, and compliance on overdiagnosis of ductal carcinoma in situ (DCIS): A modelling study
Source: PLoS One. 2026 Jan 23;21(1):e0331821. doi: 10.1371/journal.pone.0331821 (PMC12829814; doi:10.1371/journal.pone.0331821)
Supplement: S4 Table — (DOCX) [file pone.0331821.s006.docx]

**S4 Table. Screen start age and DCIS overdiagnosis rate by DCIS grade**

| Screen at age (years)^a^ | Follow-up time (years) | | | | | | | |
| --- | --- | --- | --- | --- | --- | --- | --- | --- |
|  | 2 | 3 | 4 | 5 | 10 | 15 | 20 | 25 |
| Proportion overdiagnosed (per detected DCIS in screened population) | | | | | | | | |
| DCIS Grade 1 | | | | | | | | |
| 50 | 35.9 | 32.0 | 27.9 | 24.8 | 15.3 | 11.8 | 11.3 | 11.1 |
| 52 | 46.5 | 40.0 | 36.7 | 34.0 | 20.1 | 17.3 | 16.1 | 15.7 |
| 54 | 55.8 | 49.5 | 42.8 | 38.8 | 28.9 | 23.7 | 22.0 | 21.3 |
| 56 | 46.5 | 41.5 | 37.4 | 32.9 | 23.3 | 19.8 | 18.6 | 17.6 |
| 58 | 41.2 | 36.4 | 32.8 | 30.8 | 22.3 | 18.9 | 17.9 | 17.2 |
| 60 | 36.7 | 34.1 | 29.9 | 27.9 | 19.5 | 17.8 | 16.0 | 15.7 |
| 62 | 43.9 | 39.6 | 34.4 | 32.0 | 22.8 | 20.4 | 19.1 | 19.1 |
| 64 | 40.8 | 37.1 | 33.9 | 30.7 | 23.3 | 20.0 | 19.4 | 18.9 |
| 66 | 40.9 | 36.3 | 32.9 | 29.6 | 22.5 | 19.6 | 19.0 | 18.8 |
| 68 | 43.1 | 38.7 | 34.9 | 32.7 | 25.1 | 22.7 | 21.6 | 21.6 |
| 70 | 44.5 | 40.3 | 36.3 | 33.5 | 25.3 | 23.1 | 22.6 | 22.6 |
| 72 | 47.8 | 43.8 | 38.3 | 35.1 | 27.0 | 24.0 | 23.5 | 23.5 |
| 74 | 49.4 | 43.3 | 40.0 | 36.5 | 29.2 | 25.7 | 25.5 | 25.5 |
| DCIS Grade 2 | | | | | | | | |
| 50 | 43.4 | 35.7 | 29.0 | 27.0 | 17.5 | 14.9 | 14.4 | 14.1 |
| 52 | 48.5 | 42.6 | 37.8 | 34.0 | 22.2 | 19.0 | 18.2 | 17.6 |
| 54 | 68.2 | 59.7 | 52.8 | 45.1 | 29.3 | 25.6 | 24.2 | 23.9 |
| 56 | 60.9 | 51.9 | 45.5 | 39.4 | 27.7 | 24.4 | 23.6 | 23.0 |
| 58 | 55.2 | 46.6 | 41.5 | 37.7 | 23.4 | 20.2 | 19.7 | 19.5 |
| 60 | 54.7 | 47.7 | 40.4 | 36.5 | 26.3 | 23.1 | 21.8 | 21.8 |
| 62 | 56.5 | 48.8 | 43.3 | 38.3 | 25.3 | 22.8 | 22.1 | 22.1 |
| 64 | 65.8 | 56.1 | 49.4 | 44.4 | 31.2 | 27.7 | 26.4 | 25.8 |
| 66 | 68.5 | 59.4 | 52.5 | 47.0 | 35.4 | 31.6 | 31.1 | 30.7 |
| 68 | 73.8 | 64.7 | 57.8 | 51.1 | 38.7 | 34.5 | 34.0 | 34.0 |
| 70 | 79.7 | 69.0 | 62.5 | 55.0 | 39.4 | 36.1 | 35.5 | 35.3 |
| 72 | 93.0 | 81.3 | 73.5 | 65.7 | 47.9 | 45.5 | 45.4 | 45.4 |
| 74 | 99.5 | 86.4 | 77.0 | 68.0 | 52.6 | 49.6 | 48.6 | 48.6 |
| DCIS Grade 3 | | | | | | | | |
| 50 | 44.5 | 39.3 | 33.4 | 28.6 | 17.2 | 14.0 | 13.0 | 12.6 |
| 52 | 55.4 | 45.9 | 40.5 | 36.2 | 22.7 | 17.9 | 16.4 | 16.0 |
| 54 | 70.0 | 60.2 | 52.1 | 44.7 | 28.7 | 24.8 | 23.9 | 23.8 |
| 56 | 68.4 | 56.3 | 49.9 | 43.8 | 28.3 | 24.8 | 24.4 | 24.1 |
| 58 | 64.1 | 53.4 | 45.8 | 39.9 | 25.2 | 22.0 | 21.8 | 21.7 |
| 60 | 64.0 | 54.1 | 46.3 | 41.2 | 29.6 | 27.2 | 26.5 | 26.3 |
| 62 | 61.9 | 51.7 | 44.9 | 40.5 | 29.3 | 25.1 | 24.1 | 24.0 |
| 64 | 67.3 | 57.8 | 51.0 | 44.7 | 31.7 | 26.4 | 25.6 | 25.3 |
| 66 | 74.8 | 63.7 | 56.2 | 50.5 | 33.8 | 29.6 | 28.5 | 28.5 |
| 68 | 72.5 | 63.4 | 55.5 | 48.3 | 31.5 | 28.0 | 27.2 | 27.2 |
| 70 | 82.6 | 70.7 | 60.8 | 53.6 | 38.1 | 35.7 | 35.0 | 35.0 |
| 72 | 86.1 | 72.5 | 63.9 | 57.8 | 42.7 | 39.5 | 38.8 | 38.8 |
| 74 | 84.6 | 73.1 | 60.5 | 54.7 | 41.5 | 38.2 | 38.0 | 38.0 |

Proportion overdiagnosed DCIS after a single screen at age 50-74 years for a follow-up time

of 2 to 25 years in Dutch screening setting (biennial mammography, 76% compliance).
