# Supplementary material for: Influence of follow-up, screening age, interval, and compliance on overdiagnosis of ductal carcinoma in situ (DCIS): A modelling study
Source: PLoS One. 2026 Jan 23;21(1):e0331821. doi: 10.1371/journal.pone.0331821 (PMC12829814; doi:10.1371/journal.pone.0331821)
Supplement: S5 Table — (DOCX) [file pone.0331821.s007.docx]

**S5 Table. Screening interval and compliance and DCIS overdiagnosis**

| Screen interval (years)^a^ | Compliance (%) | | | | | | |
| --- | --- | --- | --- | --- | --- | --- | --- |
|  | 50 | 60 | 70 | 80 | 90 | 100 | 76 |
| *Overdiagnosis rate (per 100,000 screened women)* | | | | | | | |
| 1 | 30.8 | 27.7 | 25.0 | 22.9 | 21.1 | 19.6 | 23.6 |
| 2 | 45.3 | 42.2 | 39.6 | 37.3 | 35.3 | 33.5 | 38.1 |
| 3 | 52.5 | 50.5 | 48.4 | 46.2 | 44.3 | 42.4 | 47.0 |
| 4 | 59.2 | 57.1 | 55.2 | 53.4 | 51.9 | 50.1 | 53.9 |
| 5 | 57.3 | 55.4 | 54.3 | 52.9 | 52.0 | 51.0 | 53.4 |

DCIS overdiagnosis rate per 100,000 screened women aged 50-74 with screening interval of

1 to 5 years for varying compliance of 50-100% and 76% of the Dutch screening setting.
