# Supplementary material for: Influence of follow-up, screening age, interval, and compliance on overdiagnosis of ductal carcinoma in situ (DCIS): A modelling study
Source: PLoS One. 2026 Jan 23;21(1):e0331821. doi: 10.1371/journal.pone.0331821 (PMC12829814; doi:10.1371/journal.pone.0331821)
Supplement: S6 Table — (DOCX) [file pone.0331821.s008.docx]

**S6 Table. Sensitivity analysis: exclusion of direct progression to IBC**

| Population size^a^ | Total | DCIS grade | | |
| --- | --- | --- | --- | --- |
|  |  | 1 | 2 | 3 |
| Overdiagnosis rate (per 100,000 screened women) | | | | |
| 100% | 38.1 | 8.8 | 14.7 | 14.7 |
| 82% | 38.6 | 9.0 | 14.4 | 15.2 |
| Proportion overdiagnosed (per detected DCIS in screened population) | | | | |
| 100% | 19.6% | 23.6% | 20.0% | 17.5% |
| 82% | 19.9% | 24.3% | 19.8% | 18.1% |

DCIS overdiagnosis rate (per 100,000 women screened) and proportion overdiagnosed DCIS total and stratified by DCIS grade for 82% and 100% of the population in Dutch screening setting (biennial mammography, 76% compliance). This sensitivity analysis was conducted to estimate the effect of excluding direct progression to invasive breast cancer, estimated at 18%.
