# Supplementary material for: Influence of follow-up, screening age, interval, and compliance on overdiagnosis of ductal carcinoma in situ (DCIS): A modelling study
Source: PLoS One. 2026 Jan 23;21(1):e0331821. doi: 10.1371/journal.pone.0331821 (PMC12829814; doi:10.1371/journal.pone.0331821)
Supplement: Attachment — Submitted filename: Review 1.docx [file pone.0331821.s009.docx]

**Dear Editor,**

I read with interest the article entitled *“Influence of follow-up, screening age, interval, and compliance on overdiagnosis of ductal carcinoma in situ (DCIS): a modelling study.”* The study addresses an important topic; however, I would like to highlight the following observations and suggestions that require clarification before the manuscript may be considered for publication:

1. Overall, the manuscript is well written and provides an evaluation of factors in breast cancer screening that may contribute to the overdiagnosis of DCIS.
2. The title is concise, accurate, and appropriate for the study.
3. The abstract adequately summarizes the manuscript, covering the background, methodology, results, and conclusions.
4. The introduction is clearly structured and appropriate.
5. In the *Materials & Methods* section, the screening age and frequency appear to have been chosen arbitrarily and do not correspond with the recommendations of either the American Cancer Society or the European Commission Initiative on Breast Cancer (ECIBC).
6. Biennial screening is not endorsed by these societies, and higher compliance rates are generally not sustainable over long-term follow-up.
7. While patients with a positive family history may require earlier and more frequent screening, the incidence of DCIS in this subgroup is relatively low.
8. The imaging modality used for screening (mammography, ultrasonography, or MRI) has not been specified. As this significantly impacts the likelihood of overdiagnosis, it should be clarified.
9. I am unable to comment on the SimDCIS tool.
10. The discussion is generally appropriate; however, it should also address the interventions and the gold standard histopathological methods required to confirm a diagnosis of DCIS.
11. The conclusion is consistent with the study findings.
12. The references are recent and relevant.

**Sincerely,**
Dr. Gyan Chand
Professor of Endocrine Surgery
SGPGIMS, Lucknow
