# Supplementary material for: Influence of follow-up, screening age, interval, and compliance on overdiagnosis of ductal carcinoma in situ (DCIS): A modelling study
Source: PLoS One. 2026 Jan 23;21(1):e0331821. doi: 10.1371/journal.pone.0331821 (PMC12829814; doi:10.1371/journal.pone.0331821)
Supplement: Attachment — Submitted filename: Response to Reviewers.docx [file pone.0331821.s010.docx]

**Response to Reviewer 1**

**1. Summary**

Thank you for taking the time to review this manuscript. Please find the detailed responses below and the corresponding revisions in track changes in the re-submitted files.

**2. Reviewer’s Responses to Questions**

1. Is the manuscript technically sound, and do the data support the conclusions?

Reviewer 1: Partly

We have addressed this point in Response 1 of the point-by-point response.

2. Has the statistical analysis been performed appropriately and rigorously?

Reviewer 1: Yes

Thank you.

3. Have the authors made all data underlying the findings in their manuscript fully available?

Reviewer 1: Yes

Thank you.

4. Is the manuscript presented in an intelligible fashion and written in standard English?

Reviewer 1: Yes

Thank you.

**3. Point-by-point response to Review Comments**

1. The main objective of the paper is to use the SimDCIS model for simulations aimed at balancing the benefits and harms of screening and avoiding overdiagnosis. However, the only significant conclusion drawn was that, in order to reduce overdiagnosis of DCIS, at least 20 years of follow-up are required. This does not appear to offer any major improvement to the current screening system, and the usefulness of the evaluation framework is questionable.

**Response 1:** Thank you for your comments. The aim of our study was not to create a model for balancing the benefits and risks of screening or to avoid overdiagnosis. Rather, the aim was to quantify the influence of certain determinants on estimates of overdiagnosis: specifically, follow-up time, screening age, interval, and compliance. To achieve this, a microsimulation model was used. Also, we recognize the rationale of our paper could be highlighted.

We have clarified the objective and rationale of the study, with the following adjustments:

Page 3, line 52 (Introduction): “*The increased detection of DCIS has raised concerns for overdiagnosis and overtreatment related to breast cancer screening, which highlights the need for optimization, including early-detection and overdiagnosis of DCIS (3,4).*”.

Page 3, line 59 (Introduction): “*This large uncertainty in estimates complicates optimization of screening, as the actual impact of DCIS overdiagnosis as major harm cannot be accurately stated.*”.

Page 4, line 85 (Introduction): “*Improved knowledge on the influence of each determinant on DCIS overdiagnosis estimates can help to understand the previously reported wide range of 20-91% overdiagnosed DCIS and improve accuracy of these estimates. These more accurate overdiagnosis estimates can be used to optimize breast cancer screening and treatment policy by making it feasible to weigh the benefit of early detection and the harm of DCIS overdiagnosis.*”.

2. Furthermore, the authors have already submitted similar findings to another journal, which further reduces the value of publishing this paper on PLOS ONE.

**Response 2**: In this publication, we quantify the influence of follow-up time and screening setting on DCIS overdiagnosis. Accurate overdiagnosis estimates are needed to consider overdiagnosis as harm in optimization of screening. To obtain accurate estimates, we need to understand the influence of determinants on overdiagnosis estimates. Also, optimization of screening should consider both DCIS and IBC.

The other papers the reviewer might refer to that used the SimDCIS model were aimed at development of the model [1], or on systematically reviewing the current literature on overdiagnosis [2]. In addition, we have one recently published paper aimed at determining the influence of the definition of overdiagnosis and DCIS grade [3]. Furthermore, we have one paper using another model (SiMRiSc) that focused on the influence of follow-up time and age for invasive breast cancer [4]. We have not yet studied, assessed, or published estimations on overdiagnosis of DCIS as a function of follow-up time and screening setting in any other submission or publication.

[1] Poelhekken K, Dorrius MD, Dibden A, Duffy SW, van der Vegt B, de Bock GH, Greuter MJW. The natural history of ductal carcinoma in situ: development, validation, and estimated outcomes of the SimDCIS model. Breast Cancer Res Treat. 2025 May;211(1):223-231. doi: 10.1007/s10549-025-07639-0.

[2] Poelhekken K, Lin Y, Greuter MJW, van der Vegt B, Dorrius MD, de Bock GH. The natural history of ductal carcinoma in situ (DCIS) in simulation models: A systematic review. Breast. 2023 Oct;71:74-81. doi: 10.1016/j.breast.2023.07.012.

[3] Poelhekken K, Greuter MJW, Van der Vegt B, Dorrius MD, de Bock GH. Overdiagnosis of ductal carcinoma in situ by grade and definition in population-based screening: a modelling study. Breast. 2025.

[4] Ding L, Poelhekken K, Greuter MJW, Truyen I, De Schutter H, Goossens M, et al. Overdiagnosis of invasive breast cancer in population-based breast cancer screening: A short- and long-term perspective. Eur J Cancer. 2022 Sep 1;173:1–9.

To clarify the relevance of this paper in relation to previous papers, we have added the following:

Page 15, line 315 (Discussion): “*Accurate overdiagnosis estimates will enable considering overdiagnosis as harm in optimization of screening. To obtain accurate estimates, an improved understanding of the influence of determinants on overdiagnosis estimates is needed and both DCIS and IBC should be considered. Therefore, in previous studies, we have developed a model for DCIS [15], systematically reviewed existing literature and models on DCIS overdiagnosis [17], assessed the influence of the definition of overdiagnosis and DCIS grade on overdiagnosis estimates [10], and determined adequate follow-up time for IBC [12]. In addition to this, the current study focused on quantification of the influence of follow-up time and screening setting on DCIS overdiagnosis. In future research, important remaining determinants should be quantified, such as breast density and screening modality, and breast cancer screening should be optimized considering both DCIS and IBC-related overdiagnosis.*”.

To avoid duplicity, the following sentence in conclusion was adjusted:

Page 16, line 334 (Conclusion): “*Meanwhile, future research should focus on quantification of other determinants~~, such as breast density and screening modality, and~~ to enable optimization of breast cancer screening taking into account both DCIS and IBC-related overdiagnosis.*”.

**Response to Reviewer 2**

**1. Summary**

Thank you for taking the time to review this manuscript. Please find the detailed responses below and the corresponding revisions in track changes in the re-submitted files.

**2. Reviewer’s Responses to Questions**

1. Is the manuscript technically sound, and do the data support the conclusions?

Reviewer 2: Yes

Thank you.

2. Has the statistical analysis been performed appropriately and rigorously?

Reviewer 2: Yes

Thank you.

3. Have the authors made all data underlying the findings in their manuscript fully available?

Reviewer 2: Yes

Thank you.

4. Is the manuscript presented in an intelligible fashion and written in standard English?

Reviewer 2: Yes

Thank you.

**3. Point-by-point response to Review Comments**

I read with interest the article entitled “Influence of follow-up, screening age, interval, and compliance on overdiagnosis of ductal carcinoma in situ (DCIS): a modelling study.” The study addresses an important topic; however, I would like to highlight the following observations and suggestions that require clarification before the manuscript may be considered for publication:

1. Overall, the manuscript is well written and provides an evaluation of factors in breast cancer screening that may contribute to the overdiagnosis of DCIS.

**Response 1:** Thank you for outlining the strengths of our manuscript and for your helpful comments.

2. The title is concise, accurate, and appropriate for the study.

**Response 2**: Thank you.

3. The abstract adequately summarizes the manuscript, covering the background, methodology, results, and conclusions.

**Response 3**: Thank you.

4. The introduction is clearly structured and appropriate.

**Response 4**: Thank you.

5. In the Materials & Methods section, the screening age and frequency appear to have been chosen arbitrarily and do not correspond with the recommendations of either the American Cancer Society or the European Commission Initiative on Breast Cancer (ECIBC).

**Response 5**: Thank you for your remark on the Methods. We agree that a justification for the choice of base scenario should be added. We have chosen the Dutch screening setting as base scenario. The model is dependent on quality of the input data, and the Dutch screening program is a long-standing well-implemented screening program, of which high quality data is available. We acknowledge that the Dutch program does not correspond with recommendations of ACS or ECIBC. Therefore, in our analyses to quantify the influence of screening setting, we already extended the start age to 40y, and look at multiple intervals and participation rates, and all possible combinations. Also, we expect the effect of follow-up time will not change for different screening setting, as we look at the time it takes to stabilize the estimate.

We have made the following adjustments to justify the choice for base scenario:

Page 6, line 122 (Methods): “*The SimDCIS model was applied to the Dutch breast cancer screening setting as base scenario, as this is a well-implemented screening program of which high quality data is available.*”.

Page 14, line 306 (Discussion): “*Also, follow-up time needed for a reliable estimate is not expected to vary for other screening settings.*”.

6. Biennial screening is not endorsed by these societies, and higher compliance rates are generally not sustainable over long-term follow-up.

**Response 6**: Thank you for your comment. We agree that compliance highly varies, and biennial screening is not the recommended screening frequency. Because compliance rates highly vary, and are often lower than in the Dutch program, we chose to vary the participation as low as 50%. The higher compliance rates are added to see the value of participation. We believe that the findings of a lower overdiagnosis for higher compliance can also be useful to explain women the value of participating in screening. Also, as stated in Response 5, the Dutch biennial screening setting was chosen due to data quality and availability. We varied the compliance for each screening frequency, to see the effect of all combinations on overdiagnosis, to make generalizability to other programs more feasible, and to quantify the influence of many combinations seen in practice. Please see the adjustments stated in Response 5.

7. While patients with a positive family history may require earlier and more frequent screening, the incidence of DCIS in this subgroup is relatively low.

**Response 7**: Thank you for pointing this out. We focused on the general population but agree that high risk population is also important to mention.

Therefore, we added the following to our limitations:

Page 14, line 307 (Discussion): “*Third, this study focused on the general population and did not consider high-risk women. Although, high-risk women may require earlier and more frequent screening, no significant change in overdiagnosis is expected, as the incidence of DCIS in this subgroup is relatively low. Fourth, …*”.

8. The imaging modality used for screening (mammography, ultrasonography, or MRI) has not been specified. As this significantly impacts the likelihood of overdiagnosis, it should be clarified.

**Response 8**: We agree that specification of the modality is very important. We have highlighted the use of mammography as follows:

Page 6, line 124 (Methods): “*In Dutch screening setting, women aged 50-74 years are invited to a biennial examination with mammography, with a compliance of 76% in 2019 (23)*”.

9. I am unable to comment on the SimDCIS tool.

**Response 9**: Thank you for clarifying. We can assure that the SimDCIS tool has been thoroughly validated, reviewed in an earlier publication, and is suitable for this application.

10. The discussion is generally appropriate; however, it should also address the interventions and the gold standard histopathological methods required to confirm a diagnosis of DCIS.

**Response 10**: Thank you for highlighting this important topic. We agree the diagnostic and treatment interventions are important to consider, especially in light of overdiagnosis and overtreatment.

Therefore, we have added the following to the discussion:

Page 13, line 282 (Discussion): “*As DCIS is always treated upon diagnosis, DCIS overdiagnosis results in overtreatment [3,5]. The golden standard of DCIS diagnosis is histopathological confirmation using a biopsy and pathological analysis to confirm a positive mammography screening [28]. In recent literature, artificial intelligence assisted histologic diagnosis has been suggested to assist pathologists to increase diagnostic accuracy and quicken the pathological evaluation [28]. The current standard of care of treatment upon detection has been under discussion [3,5]. Randomized controlled trials have been set up to explore active surveillance as an alternative [3]. If diagnosis or treatment standards change, it will be important to assess both overdiagnosis and overtreatment.*”.

11. The conclusion is consistent with the study findings.

**Response 11**: Thank you.

12. The references are recent and relevant.

**Response 12**: Thank you for your constructive comments.

**Response to Journal Requirements**

**Additional requirements**

1.Please ensure that your manuscript meets PLOS ONE's style requirements, including those for file naming.

**Response 1:** The manuscript was adjusted when necessary to meet PLOS ONE’s style requirements, including those for file naming.

2. Please note that PLOS One has specific guidelines on code sharing for submissions in which author-generated code underpins the findings in the manuscript. In these cases, all author-generated code must be made available without restrictions upon publication of the work. Please review our guidelines and ensure that your code is shared in a way that follows best practice and facilitates reproducibility and reuse.

**Response 2:** The guidelines on code sharing for submissions were checked and followed.

3. Please provide additional details regarding participant consent. In the ethics statement in the Methods and online submission information, please ensure that you have specified (1) whether consent was informed and (2) what type you obtained (for instance, written or verbal, and if verbal, how it was documented and witnessed). If your study included minors, state whether you obtained consent from parents or guardians. If the need for consent was waived by the ethics committee, please include this information.

If you are reporting a retrospective study of medical records or archived samples, please ensure that you have discussed whether all data were fully anonymized before you accessed them and/or whether the IRB or ethics committee waived the requirement for informed consent. If patients provided informed written consent to have data from their medical records used in research, please include this information.

**Response 3:** The following ethics statement can be found in the manuscript: “*This study used publicly available fully anonymized retrospective aggregated data; no individual data was used. Our local institutional review board (IRB) confirmed this study was exempt from ethical compliance and waived the requirement for informed consent (IRB number M24.343483, Medical Ethics Review Board Groningen, The Netherlands).*”.

4. Please note that funding information should not appear in any section or other areas of your manuscript. We will only publish funding information present in the Funding Statement section of the online submission form. Please remove any funding-related text from the manuscript.

**Response 4:** All funding-related text was removed from the manuscript and added to the Funding Statement section of the online submission form.

5. If the reviewer comments include a recommendation to cite specific previously published works, please review and evaluate these publications to determine whether they are relevant and should be cited. There is no requirement to cite these works unless the editor has indicated otherwise.

**Response 5:** No specific previously published works were recommended by the reviewers.
