# Supplementary material for: Influence of follow-up, screening age, interval, and compliance on overdiagnosis of ductal carcinoma in situ (DCIS): A modelling study
Source: PLoS One. 2026 Jan 23;21(1):e0331821. doi: 10.1371/journal.pone.0331821 (PMC12829814; doi:10.1371/journal.pone.0331821)
Supplement: Attachment — Submitted filename: Response to Reviewer_submit.docx [file pone.0331821.s011.docx]

**Response to Reviewer 3**

**1. Summary**

Thank you for taking the time to review this manuscript. Please find the detailed responses below and the corresponding revisions in track changes in the re-submitted files.

**2. Reviewer’s Responses to Questions**

1. Is the manuscript technically sound, and do the data support the conclusions?

Reviewer 1: Yes

Thank you.

2. Has the statistical analysis been performed appropriately and rigorously?

Reviewer 1: Yes

Thank you.

3. Have the authors made all data underlying the findings in their manuscript fully available?

Reviewer 1: Yes

Thank you.

4. Is the manuscript presented in an intelligible fashion and written in standard English?

Reviewer 1: Yes

Thank you.

**3. Point-by-point response to Review Comments**

1. There are many definitions of overdiagnosis, the authors need to justify why they chose the one mentioned in the manuscript.

**Response 1:** Thank you for your comments. We agree the justification of the chosen definition could be clarified. Previously we have published an article on different definitions in literature and recommended a definition for DCIS overdiagnosis. This definition was used in this article.

To justify the definition, the following was added to the Methods section:

Page 6, line 131 (Methods): “*The definition of DCIS overdiagnosis varies in literature. Previous recommendations highlight that estimates of DCIS overdiagnosis should account for progression to IBC, include clinical detection, and should be made from population or individual perspective [26]. In our previous publication, a definition for DCIS overdiagnosis was recommended [10], which was also used in this study. Overdiagnosed DCIS was defined as …*”.

2. The denominator size has been influenced by excluding direct progression to IBC. The authors need to comment on the magnitude of this bias.

**Response 2**: We agree that the exclusion of direct progression influences the overdiagnosis estimate. In our limitations, we already stated that the estimation could be influenced by this exclusion, due to a larger healthy population. However, we did not quantify this effect. To explore this, we did an additional sensitivity analysis. In literature, an 18% direct progression from healthy to IBC is suggested. Therefore, we lowered our healthy population to 82% to estimate DCIS overdiagnosis. This resulted in a 1% lower estimate of DCIS overdiagnosis in Dutch setting.

To estimate the magnitude of overestimation due to exclusion of direct progression to IBC, we have added the following:

Page 6, line 114-119 (Methods): “*Direct progression from healthy to IBC was not included in SimDCIS, as the main goal of the model was to estimate DCIS overdiagnosis which is not affected by IBC without DCIS as a precursor. The exclusion of direct progression to IBC, which was estimated at 18% [20], resulted in a larger healthy population compared to real-world. To estimate the effect of this assumption on our overdiagnosis estimate, an additional sensitivity analysis was performed with 82% of the population.”.*

Page 14, line 306-311 (Discussion): “*In addition, direct progression from healthy to IBC was not included in SimDCIS, as the main goal of the model was to estimate DCIS overdiagnosis. Although DCIS overdiagnosis is not directly affected by the number of IBC without DCIS as a precursor, the population of healthy people is larger without the possibility for direct progression. Therefore, there could be bias in our estimation of DCIS overdiagnosis. Sensitivity analysis showed an underestimation of approximately 1% in Dutch screening setting (S6 Table).”.*

3. The clarity on generalizability of this study needs to be better.

**Response 3**: We agree that the paragraph on generalizability of this study could be clarified. The model was shown to be suitable in multiple screening settings. However, in this study we show overdiagnosis estimates depend on the specific screening setting. The exact overdiagnosis estimate should therefore be made for a specific setting. We estimate that the influence of each factor is similar for different screening programs, but generalizability is restricted to countries with a well-implemented screening program.

To clarify generalizability of the study, the following was added:

Page 15, line 3011-320 (Discussion): “*Second, the SimDCIS model was extensively validated previously and shown to make reliable estimates in context of screening in both Dutch and UK setting [10,15]. However, ~~the model only provides a simplification of the truth and always bound to a specific setting.~~ comparisons with other programs should be done with care, as overdiagnosis estimates should be made for a specific setting. Results of the Dutch screening setting and its variations will likely be comparable to other well-implemented screening programs but are not suited for areas without screening. The impact of a specific factor on the overdiagnosis estimate is, however, expected to be similar. Also, the estimated follow-up time needed for a reliable DCIS overdiagnosis estimate is not expected to vary for other screening settings*.”

4. In table-1 better to avoid arrows to depict the increasing and decreasing trend.

**Response 4**: We have adjusted table 1 according to this suggestion, and the arrows were removed.

**Response to Journal Requirements**

**Additional requirements**

1. If the reviewer comments include a recommendation to cite specific previously published works, please review and evaluate these publications to determine whether they are relevant and should be cited. There is no requirement to cite these works unless the editor has indicated otherwise.

**Response 1:** No recommendations for citations were done.

2. Please review your reference list to ensure that it is complete and correct. If you have cited papers that have been retracted, please include the rationale for doing so in the manuscript text, or remove these references and replace them with relevant current references. Any changes to the reference list should be mentioned in the rebuttal letter that accompanies your revised manuscript. If you need to cite a retracted article, indicate the article’s retracted status in the References list and also include a citation and full reference for the retraction notice.

**Response 2:** The reference list was reviewed. No retracted articles were found.

Two references were added to justify our response to the reviewer.

[20] Lips EH, Kumar T, Megalios A, Grand Challenge PRECISION consortium; et al. Genomic analysis defines clonal relationships of ductal carcinoma in situ and recurrent invasive breast cancer. Nat Genet. 2022 Jun;54(6):850-860. doi: 10.1038/s41588-022-01082-3.

[26] Hendrick RE, Monticciolo DL. USPSTF Recommendations and Overdiagnosis. J Breast Imaging. 2024 Jul 30;6(4):338-346. doi: 10.1093/jbi/wbae028.
